# Volume quantification by contrast-enhanced ultrasound: an in-vitro comparison with true volumes and thermodilution

**DOI:** 10.1186/1476-7120-11-36

**Published:** 2013-10-17

**Authors:** Ingeborg HF Herold, Gianna Russo, Massimo Mischi, Patrick Houthuizen, Tamerlan Saidov, Marcel van het Veer, Hans C van Assen, Hendrikus HM Korsten

**Affiliations:** 1 Department of Anesthesia and Intensive Care, Catharina hospital Eindhoven, Michelangelolaan 2, Eindhoven 5623 EJ, The Netherlands; 2 Department of Electrical Engineering, Eindhoven University of Technology, Den Dolech 2, Eindhoven 5612 AZ, The Netherlands; 3 Department of Cardiology, Catharina hospital Eindhoven, Michelangelolaan 2, Eindhoven 5623 EJ, The Netherlands

**Keywords:** Contrast-enhanced ultrasound, Thermodilution, Blood volume, Indicator-dilution curve

## Abstract

**Background:**

Contrast-enhanced ultrasound (CEUS) has recently been proposed as a minimally- invasive, alternative method for blood volume measurement. This study aims at comparing the accuracy of CEUS and the classical thermodilution techniques for volume assessment in an in-vitro set-up.

**Methods:**

The in-vitro set-up consisted of a variable network between an inflow and outflow tube and a roller pump. The inflow and outflow tubes were insonified with an ultrasound array transducer and a thermistor was placed in each tube. Indicator dilution curves were made by injecting indicator which consisted of an ultrasound-contrast-agent diluted in ice-cold saline. Both acoustic intensity- and thermo-dilution curves were used to calculate the indicator mean transit time between the inflow and outflow tube. The volumes were derived by multiplying the estimated mean transit time by the flow rate. We compared the volumes measured by CEUS with the true volumes of the variable network and those measured by thermodilution by Bland-Altman and intraclass-correlation analysis.

**Results:**

The measurements by CEUS and thermodilution showed a very strong correlation (r_s =_ 0.94) with a modest volume underestimation by CEUS of −40 ± 28 mL and an overestimation of 84 ± 62 mL by thermodilution compared with the true volumes. Both CEUS and thermodilution showed a high statistically significant correlation with the true volume (r_s =_ 0.97 (95% CI, 0.95 - 0.98; P<0.0001) and r_s =_ 0.96 (95% CI, 0.94 - 0.98; P<0.0001, respectively).

**Conclusions:**

CEUS volume estimation provides a strong correlation with both the true volumes in-vitro and volume estimation by thermodilution. It may therefore represent an interesting alternative to the standard, invasive thermodilution technique.

## Background

Blood volume determination is a daily routine in anesthesia and intensive care practice. Most of the time, it is roughly estimated using clinical parameters such as blood pressure, heart frequency, urine output, and peripheral temperature. In sepsis, the postoperative phase and heart failure, circulating volume can be difficult to assess and in these cases classical dilution techniques are of additional value**.** The intrathoracic blood volume can be estimated by transthoracic thermodilution, presently one of the most widely used techniques. Its value and change in response to fluid challenge reflects the left ventricular preload and changes in preload better than more conventional measures like central venous pressure and pulmonary artery wedge pressure [1]. However, these techniques are invasive and require catheterization of the heart [2] and/or large vessels [3], which can lead to complications.

With classical dilution techniques, a known amount of indicator is injected via a central venous line into the jugular or subclavian vein and is carried through the heart and pulmonary circulation where it is mixed and diluted. Downstream, the indicator concentration-change over time is measured at a detection site to create an indicator dilution curve (IDC) [4]. The IDC is used to estimate the mean transit time (MTT); this is the average time it takes for the indicator to travel from the injection site to the detection site [5]. When two detection sites are used, the product of MTT difference and flow can be used to calculate the volume in between both sites. Classical indicator-dilution techniques can be performed with different standard indicators (such as cold saline, indocyanine green or lithium) through different access sites (e.g. right or left heart-sided) [2-4,6]. The transpulmonary thermodilution technique allows measurement of cardiac output (CO) and intrathoracic blood volumes [3,6].

A less invasive technique may be a valuable alternative to these methods, which are hampered by their invasiveness. A promising minimally invasive alternative technique uses an ultrasound contrast agent (UCA) injected into a peripheral vein as indicator. This can be detected noninvasively by contrast-enhanced ultrasound (CEUS) imaging. Mischi et al. previously demonstrated that this technique can be used for estimating blood volumes [7-9]. In this study the measurement of blood volumes by means of UCA dilution with transthoracic echography (TTE) was tested and validated in-vitro. The determination coefficient between the real and the estimated volumes was larger than 0.999 in different model fits [7].

However, to date there has been no comparison with the classic thermodilution technique, which is clinically considered the gold standard for CO and blood volume measurement.

The aim of our study is to compare the CEUS with the thermodilution technique for volume quantification in an in-vitro set-up with different flows and volumes. We decided to use the transesophageal probe as this probe is often used in the perioperative setting, where large volume shifts can occur.

## Methods

### In-vitro set-up

**Figure 1:**
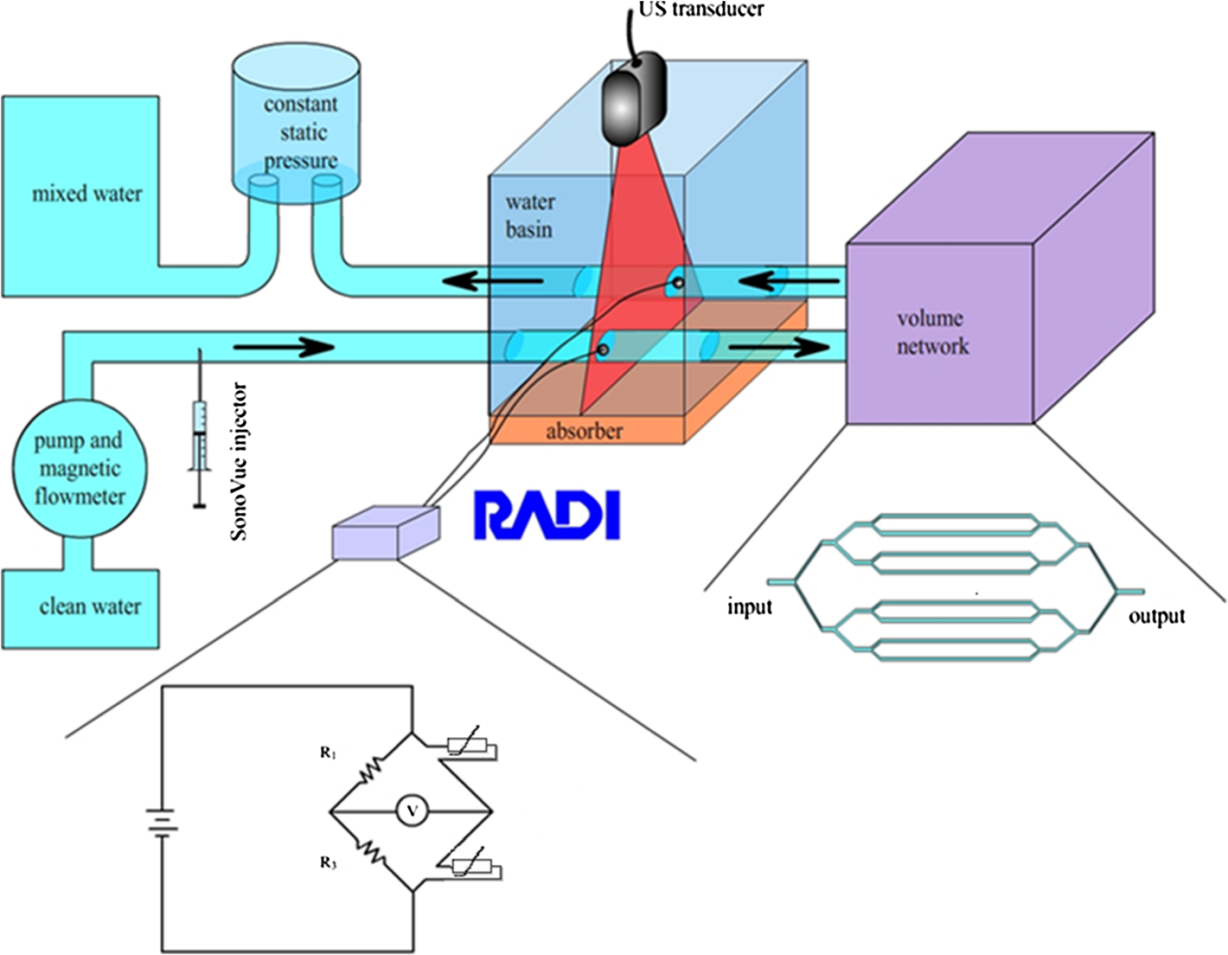
**The in-vitro set-up in a schematic overview.** The variable network can be clamped at different points to create different volumes.

The realized in-vitro set-up (Figure 1) consisted of an open circuit with a roller pump, Cobe Stoeckert multiflow bloodpump (Stoeckert Instruments, Munich, Germany), a water-filled basin, a network of tubes with a variable volume simulating the pulmonary vessels, and a pressure stabilizer. The whole set-up was filled with tap water which was degassed by 24-hour rest. The temperature was maintained at 37°C with heating devices and thermostats at different positions in the set-up. The in- and outflow tubes of the network were submerged in a water-filled basin. The submerged segment of the tubes was made of a thin polyurethane layer (Ultracover®, Microtek™ Medical BV, Zutphen, the Netherlands) in order to limit interference with ultrasound measurements. In the water-filled basin, a transesophageal (TEE) probe (X7-2 t, Philips Healthcare, MA, USA) was directly submerged in water to optimize the acoustic impedance while insonifying the submerged tubes. Two 0.014” high-fidelity pressure wires (Radiwire, St Jude Medical Inc, St. Paul, MN, USA) were inserted in these tubes. These wires measure temperature at 0–25 Hertz (Hz) with an accuracy of 0.05°C within a temperature range of 15 to 42°C. Distal to the centrifugal pump, cold saline and UCA were injected into the inflow tube through an injection point consisting of a single lumen central venous line (Blue flextip catheter, Arrow®, Reading, PA, USA). Between the inflow and outflow tubes, outside the basin, the circuit expanded into a network of eight tubes and converged back into a single outflow tube. This network was made of tubing which is used for cardiopulmonary bypass, matching the roller pump. The tubing (Medtronic, Minneapolis, MN, USA) of the network had a diameter of ¼” with a wall size of ^3^/_32_”. The afferent and efferent tubes had a diameter of ½”. The length of tubing was adapted to create a physiologic range of volumes [10,11]. The network could be clamped at different positions to create different volumes. The hydrodynamic circuit was open to avoid UCA recirculation and the hydrostatic pressure of the circuit was stabilized at the output. All tubes were isolated with polyethylene covers (Climaflex®, NMC, Eynatten, Belgium) to prevent temperature loss to the surroundings.

### Ultrasound system and settings

A commercially available scanner (iE33, Philips Healthcare, Andover, MA, USA) was used to obtain cross-sectional B-mode images of the inflow and outflow tubes. Harmonic imaging at 2.7 - 5.4 MHz was used in order to increase the signal-to-noise ratio (SNR) for low UCA concentration together with a low mechanical index (MI) of 0.2 to reduce bubble disruption. Frame rate was set at 27 Hz, the same time-gain and lateral-gain compensation were employed over all measurements, compression was set at 50 dB, general gain at 60%, and image depth was 8 cm with the focus being at the level of both tubes.

### Calibration

**Figure 2:**
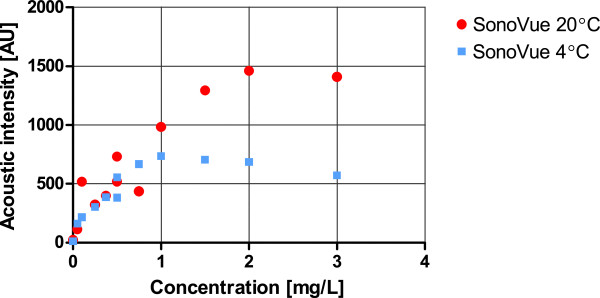
**Acoustic intensity calibration curves of SonoVue® at room temperature and at a temperature <4°C.** Acoustic intensity is presented on the Y-axis at different temperatures and at different concentrations of SonoVue®. At room temperature (red circle) there is attenuation above 1.5 mg/L; at a temperature <4°C (light blue square) attenuation occurs at a concentration between 1 mg/L and 1.5 mg/L. A linear relationship between concentration and acoustic intensity is seen below 1 to 1.5 mg/L at both temperatures.

For direct application of the indicator dilution theory, a linear relationship between UCA concentration and detected acoustic intensity is necessary [8]. Therefore, we measured the different acoustic intensities of different doses of UCA (SonoVue®, Bracco SpA, Geneva, Italy) diluted in saline at room temperature and at 4°C. This calibration was performed according to the protocol described by Mischi et al. [7]. It had a twofold objective: finding the range of UCA concentrations that show a linear relationship with the measured acoustic intensity, and investigating the effect of temperature on the UCA behavior. The relationship between SonoVue®-concentration and measured acoustic intensity was linear below 1.5 mg/L (Figure 2) at room temperature and 1 mg/L at 4°C. Above these concentrations shadowing was seen.

### Thermodilution measurement

Thermodilution measurements were performed using the pressure wires as described above. These pressure wires have temperature sensing tips that were positioned in the polyurethane tubes and were intercepted by the ultrasound beam for contrast quantification. The temperature sensors of both pressure wires were connected to a Wheatstone bridge adjusted to half-bridge configuration in order to output measured IDCs from both sensors. The electrical circuit further comprised a feedback amplifier (INA 118, Burr-Brown Corporation, Tucson, AZ, USA), a power supply (Delta Elektronica, Zierikzee, the Netherlands), and a data acquisition board (NI USB-6341, National Instruments, Austin, TX, USA). The bridge was balanced by manual adjustment of the value of an embedded potentiometer. The output signal was amplified in such a way that the full range of the analog-to-digital converter of the data acquisition card (0-10 V) was exploited. High frequency noise suppression was achieved by placing an additional capacitance in parallel with the input impedance of the amplifier. All devices were shielded and grounded to minimize ambient disturbances. The thermodilution curves were acquired with LabVIEW (National Instruments, Austin, TX, USA) and processed in MATLAB® 2009b (The Mathworks, Natick, MA, USA). The full system was calibrated by mapping the measured voltage as a function of temperature in a water-filled basin measured by a digital thermometer (Keithley 871, Keithley Instruments, Cleveland, OH, USA). The calibration showed a linear relationship with a slope of 0.65 V/°C and r^2^ = 0.999. These results confirmed the system linearity for temperatures in a range 24°C – 40°C.

### Ultrasound contrast measurement

Different flows were generated by adjusting the rounds per minute (rpm) of the centrifugal pump. Six flows were used for the measurements that varied between 1 and 4 liters per minute in increments of 0.5 liter per minute. Flow was measured using a flow sensor (Flow controller ARS 260, Biotech, Vilshofen, Germany), at the end of the circuit. By clamping different bifurcations of the variable network, four different volumes were generated, namely 890 milliliter (mL), 718 mL, 530 mL, and 356 mL (Figure 1). These volumes have been chosen to cover a range that is slightly broader than the pulmonary blood volumes reported in patients, which range from 271 mL/m^2^ (~500 mL) to 421 mL/m^2^ (~800 mL) in heart failure patients [10,11]. Every measurement was repeated three times, at six flows and four volumes. With every measurement a bolus of 0.2 mL SonoVue® diluted in 20 mL cold saline (4°C) was injected. The change in acoustic intensity on B-mode ultrasound was stored in an uncompressed format for subsequent analysis with commercially available software (QLAB 8, Philips Healthcare, Andover, MA, USA). This software allows drawing of multiple regions of interest (ROIs) to obtain acoustic IDCs. Two ROIs were drawn within the thin polyurethane layer of the inflow and outflow tube in the water-filled basin. An additional movie file shows this in more detail (see Additional file 1). The IDCs were processed and fitted by the local density random walk (LDRW) model using MATLAB® 2009b [12]. The LDRW model was employed since it provides both the best least square error fit to the IDC and a physical description of the dilution process. The MTT of the contrast bolus between the injection and the detection sites was directly derived from the parameters of the fitted model [7]. Volumes were then calculated as the product between the measured flow and the difference in MTT between the two curves.

**Figure 3:**
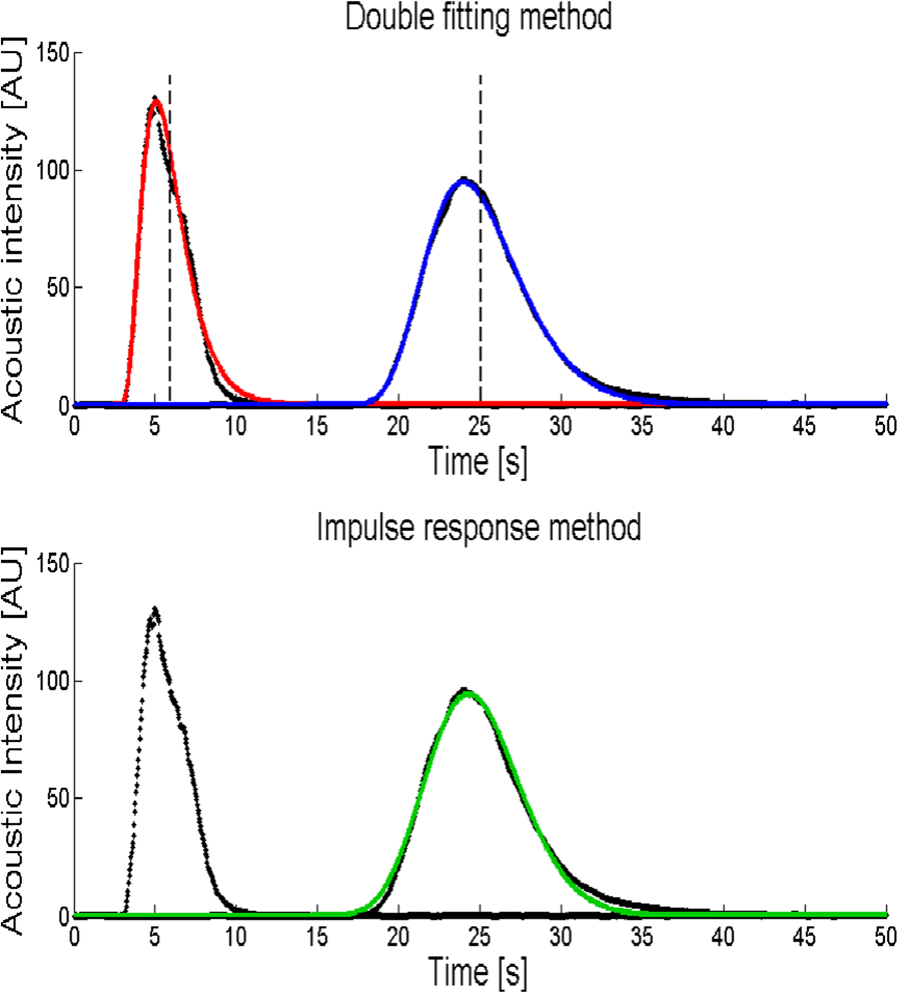
**Indicator dilution curves (IDCs) fitted by LDRW double fit method and impulse response method.** These IDCs were constructed from post-processing imaging analysis at the level of inflow and outflow tubes at a flow of 2 L/min and true volume of 718 mL. The dotted black line is the IDC at the level of the inflow tube and outflow tube. In the upper picture, the red and blue lines depict the IDCs by the double fit method according to the LDRW model of the inflow and outflow tube. The vertical dashed lines represent the MTTs of each curve. In the bottom picture, the green line depicts the IDC according to the LDRW model impulse response method.

The MTT can be derived using two different methods. First, the MTT of each IDC can be estimated as the first order statistical moment of the fitted model, using the double fit method (Figure 3). Second, the indicator dilution system can also be interpreted as a linear system; therefore, the impulse response approach can be employed [7,8]. The impulse response of the system between the two indicator detection sites was estimated by means of a parametric deconvolution technique, using the system input and output signals represented by the measured IDCs [8]. The estimated impulse response is represented by the LDRW model, which allows blood volume assessments (Figure 3). The advantage of using a deconvolution technique over a double IDC fitting consists of the independency of the resulting impulse response from the injection function [7].

### Statistics

All data were reported as mean values ± standard deviation (SD) or as median ± interquartile range (IQR) depending on the distribution of the variables of three consecutive measurements. The first goal was to investigate the agreement between measured volumes by both techniques and the true set-up volumes. Statistical significance was considered as a two-sided P<0.05. Bland-Altman analysis was used to determine the agreement between measured volumes and the true volumes [13]. The effect of the different flows on the volume measurement was also investigated and reported in dedicated plots. Reproducibility was assessed by the intraclass-correlation coefficient (ICC). ICC consists of a basic calculation as repeated-measures analysis of variance (ANOVA) and the intraobserver reliability (ICC (1,1)). ICC assesses the agreement of quantitative variables on its reliability and consistency [14,15]. The second goal was to analyze the correlation between the CEUS volumes and thermodilution volumes, assuming thermodilution as the gold standard. Correlation coefficients were assessed using the Pearson correlation coefficient R or the Spearman correlation coefficient r_s_ depending on normal distribution or non-normal distribution of variables, respectively. Statistical analysis was performed using GraphPad Prism version 5.03 (GraphPad Software, San Diego, CA, USA) except for the intraclass-correlation, which was analyzed by Unistat® Statistical Package for Windows™ version 6.0 (Unistat House, London, England). Statistical analysis was performed by using all data (n=72 measurements) to exclude a bias.

## Results

### Measurements

A total of 79 measurements were performed. Seven measurements could not be used for analysis as a result of failed acquisition on the ultrasound equipment (n=5) or due to technical failure of the Wheatstone bridge (n=2). All remaining 72 measurements were used for analysis. The CEUS derived median volume of these, using the LDRW model double fit method was 590 (394–764) mL. For the impulse response method, the volume was 574 (382 –725) mL. The median volumes estimated with the thermodilution technique double fit method and impulse response method were 722 (489 – 944) mL and 693 (459 – 886) mL, respectively.

### Reproducibility

Repeated-measures ANOVA demonstrated no significant variance between the measures for both CEUS and thermodilution-derived volumes. Intraclass-correlation between three repetitive measurements was ICC = 0.99 (95% confidence interval (CI), 0.98 - 1.00) for the CEUS derived volumes and ICC = 0.97 (95% CI, 0.94 - 0.98) for the thermodilution calculated volumes, using the double fit method. The intraclass-correlation for the measured volumes using the impulse response method was ICC = 0.98 (95% CI, 0.96 - 0.99) for CEUS and ICC = 0.98 (95% CI, 0.97 - 0.99) for thermodilution.

### Effect of flow and volume on the measurements

**Figure 4:**
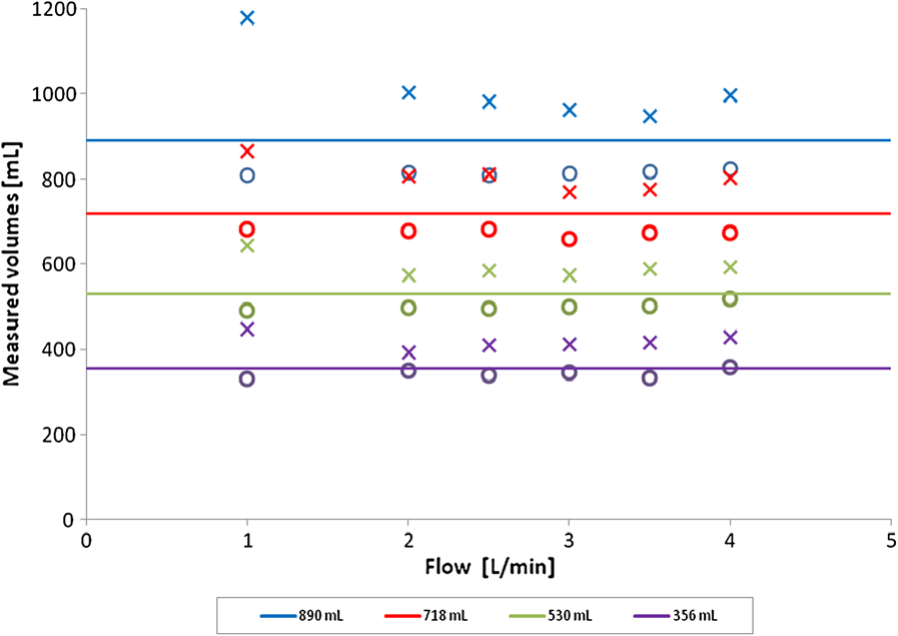
**Volume measurements using contrast enhanced ultrasound (bullets) or thermodilution (crosses) at different flow rates and volumes.** The different volume settings (true volumes) are indicated in the legend and expressed in the graph as solid lines. Each volume measurement is the average of three repetitions.

With the set-up completely open (largest volume, 890 mL), the CEUS-derived volumes, averaged over all flows, were underestimated by −74 mL, for a true volume of 718 mL the underestimation was −43 mL, for 530 mL it was −30 mL, and for 356 ml it was −13 mL. Using thermodilution on the other hand, a general overestimation was seen. For 890 mL the average overestimation was +122 mL, for 718 mL it was +88 mL, for 530 mL it was + 64 mL, and for 356 mL it was + 63 mL. All the volumes were measured by the LDRW double fit method. In both CEUS and thermodilution, the deviations with respect to the true volumes were larger at larger volumes (Figure 4).

### Correlation between the measured volumes and true volumes

**Figure 5:**
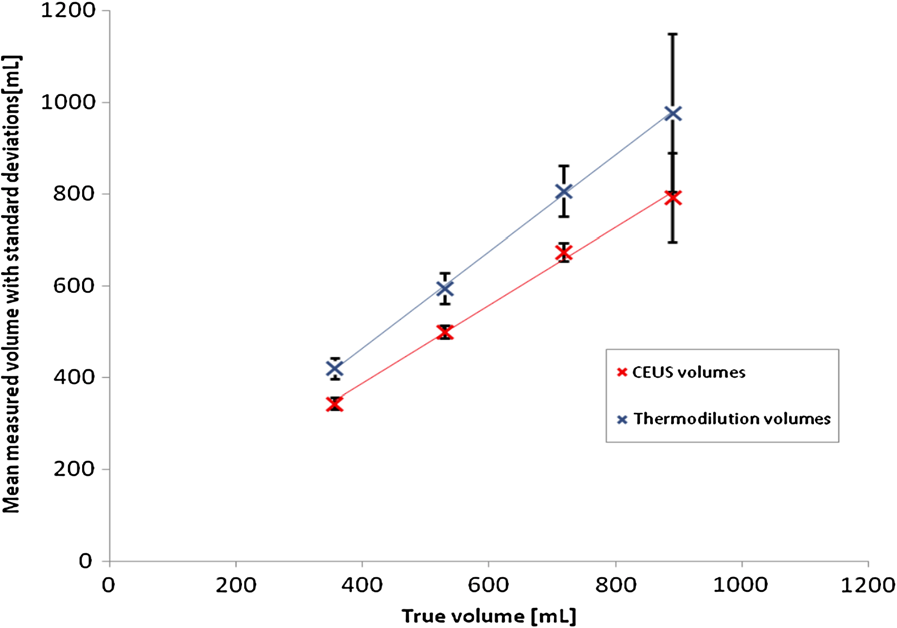
**Correlation with standard deviation is shown between the mean volumes measured by contrast-enhanced ultrasound or thermodilution and true volumes.** For volume measurements the LDRW double fit method was used. Linear regression analysis revealed following trend: Mean CEUS volume = 0.85 true volume + 47 (r^2^ = 0.996). Mean thermodilution volume = 1.05 true volume + 42 (r^2^ = 0.999).

**Figure 6:**
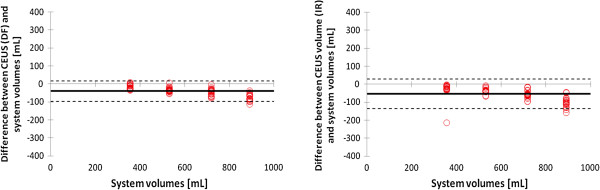
**Bland-Altman plots showing the agreement between measured volumes by contrast enhanced ultrasound and true volume of the set-up.** The volumes measured with contrast enhanced ultrasound are displayed in left and right panel, using the LDRW double fit (DF) method and LDRW impulse response (IR) method, respectively. The bold line indicates the mean difference between CEUS measured and true volumes (bias), the dashed lines indicate two times the standard deviation.

**Figure 7:**
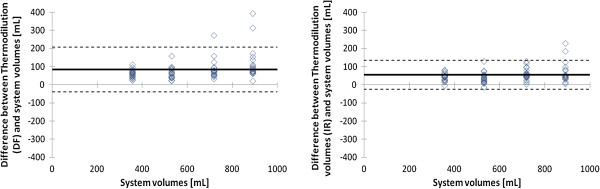
**Bland-Altman plots showing the agreement between measured volumes by thermodilution technique and true volumes of the set-up.** The volumes measured with thermodilution technique are indicated in left and right panel, using the LDRW double fit (DF) method and the LDRW impulse response (IR) method, respectively. The bold line indicates the mean difference between CEUS measured and true volumes (bias), the dashed lines indicate two times the standard deviation.

The correlation between the 72 volumes measured with CEUS and the true volumes showed r_s_ = 0.97 (95% CI, 0.95 - 0.98; P<0.0001) using the LDRW double fit method and r_s_ = 0.97 (95% CI, 0.95 - 0.98; P<0.0001), using the LDRW impulse response method. The correlation for the 72 measured volumes using the thermodilution technique showed r_s_ = 0.96 (95% CI, 0.94 - 0.98; P<0.0001) using the LDRW double fit method. When the LDRW impulse response method was used for the thermodilution measured volumes r_s_ = 0.97 (95% CI, 0.95 - 0.98; P<0.0001). Figure 5 shows the linear regression analysis for volumes measured with CEUS and the LDRW double fit method. All measured volumes correlated significantly with the true volumes. Bland-Altman analysis [13] (Figure 6) demonstrated a bias between CEUS and true volumes of −40 ± 28 mL using the LDRW double fit method and −53 ± 41 mL using the LDRW impulse response method. The bias of the thermodilution volumes compared to the true volumes was 84 ± 62 mL and 55 ± 40 mL, for the double fit and impulse method respectively (Figure 7).

### Correlation between volumes estimated with CEUS and the thermodilution technique

The correlation between CEUS and thermodilution technique showed r_s_ = 0.94 (95% CI, 0.90 - 0.96; P<0.0001), using the LDRW double fit method and r_s_ = 0.97 (95% CI, 0.95 - 0.98; P<0.0001), using the impulse response method. The Bland-Altman analysis showed a bias of −124 ± 74 mL using the LDRW model double fit method, where thermodilution estimated volumes were on average larger than CEUS volumes. For the LDRW model impulse response method the bias was −108 ± 67 mL.

## Discussion

In the present study, comparing volume measurement by CEUS with thermodilution in a controlled in-vitro set-up, we have demonstrated that there is a good correlation between volumes measured by CEUS and by thermodilution (r_s_ = 0.94 with the LDRW double fit method). Using the Bland-Altman analysis, there was a good level of agreement (bias −108 ± 67 mL with the LDRW double fit method) between both methods with only a modest underestimation of the true volume by CEUS (bias −40 ± 28 mL with the LDRW double fit method). Interestingly, compared to the gold standard of thermodilution, CEUS demonstrated to provide a more accurate measure of a known volume in this in-vitro set-up with a bias of only −40 ± 28 mL compared to 84 ± 62 mL for the thermodilution method.

In general, the thermodilution technique overestimated all volumes (Figure 4). The overestimation of the thermodilution volumes can be explained, in the first place, by loss of heat to the surroundings due to conduction across the tube wall. Heat loss is more marked at low flows and large volumes (Figure 4) due to longer contact-time and larger surface area, respectively. In order to minimize this effect, we isolated the whole set-up and the temperature was kept stable in a narrow range around 37°C. Despite this, the volume overestimation was consistently present even at small volumes (356 mL) and high flows (4 liters per minute), when heat loss is expected to be minimal.

In our study, we used a TEE matrix probe, as this is a more realistic set-up to perform volume measurements by CEUS during open heart surgery and the perioperative phase. In general the proposed methods are also feasible by TTE, as shown in studies by Mischi [7,8]. In comparison to TTE, TEE is closer to the heart with minimal ultrasound attenuation in between. Whether the results with a TEE or TTE probe are interchangeable needs to be investigated in future research.

As no calibration is available for the adopted TEE probe in literature, we calibrated the probe by determining the relationship between the SonoVue® concentration and measured acoustic intensity. The calibration was performed at two different temperatures, namely ambient room temperature (20°C) and the typical temperature used for cold thermodilution (4°C). To this purpose, we diluted SonoVue® in saline at 4°C to obtain sufficient signal-to-noise ratio for the thermodilution IDC and because this temperature is routinely used for clinical thermodilution measurements. We found UCA bubbles to be stable longer at lower temperatures [16], however at the cost of a reduced echogenicity (Figure 2). This is in line with the reported decrease in bubble stability at higher temperatures [17,18]. At higher temperature the UCA bubbles expand 5% by gas expansion and show increased acoustic backscatter [19]. Our calibration showed no attenuation below 1.0 mg/L at 4°C. Higher doses will produce a non-stationary (concentration dependent) shadow effect that will influence the IDC quantification, possibly affecting the MTT. We calculated our diluting volume for SonoVue® as the total volume in the circuit, which was over 1 liter. Nevertheless at the lowest volume of the tube network (356 mL) and highest flow (4 L/min) we registered some attenuation in the IDC, probably due to a low effective diluting volume of less than 1 L. In spite of this, the MTT reproducibility at these settings was high with an intraclass-correlation of ICC = 0.99 and an average difference with the true volumes of +2 mL for CEUS and for thermodilution +74 mL (Figure 4). Therefore, the influence of attenuation on the MTT assessment seems negligible.

The IDCs are fitted using dedicated models that can possibly influence volume measurement thus leading to over- and/or underestimation. With this respect, the LDRW model seems superior as the fitting is based on both the ascending and descending slopes of the IDC which makes it less sensitive to noise. Still, this may lead to underestimation of the IDC tail compared to more common models like mono-exponential and power-law model [5]. In a study of Ugander et al. magnetic resonance imaging was evaluated as a method for estimating pulmonary blood volume [20]. An in-vitro validation of pulmonary blood volume measurements was carried out, showing a mean difference between the measured volumes and true volumes of 10 ± 2% for the peak-to-peak method and 4 ± 3% for the center of gravity method [20]. The center of gravity and peak-to-peak methods do not use model fitting for the MTT estimation, and they are more sensitive to low signal-to-noise ratios and contrast recirculation. Moreover, they do not provide a physical interpretation of the investigated convective diffusion process, as provided by the LDRW model [21]. The LDRW model provides better fits of skewed IDCs [5], which are present at high flows and small volumes. Our mean difference for CEUS and the true volumes was −6.5 ± 2.8% using the LDRW double fit method and −8.5 ± 2.9% using LDRW impulse response method. A volume underestimation of −3.3 ± 2.3% was also found by different models and settings in another in-vitro study for volume quantification with magnetic resonance imaging [22]. In particular a slightly lower accuracy and volume underestimation by the impulse response method was reported; however, this was not confirmed by intra-thoracic blood volume measurements in the volunteers. The advantage of the impulse response method consists of making the measurement robust to recirculation and variations in the injection function. Moreover, the identification of the full transpulmonary dilution impulse response brings additional information, possibly adding diagnostic value to the analysis [22].

Another finding to be discussed relates to the volume underestimation by CEUS. This is likely to be explained with the bubble transport kinetics. It has been reported that bubbles, especially for laminar flow (Reynolds < 2000), show a velocity profile that differs from that of the carrier fluid, leading to a shorter MTT. In particular, while the carrier fluid shows a typical parabolic flow profile, bubbles are reported to travel with a “flatter” profile, whose average velocity over the tube cross section is higher than that of the carrier fluid [23,24]. Additional explanations, to be verified in future studies, might relate to the concentration profile of bubbles across the tube.

In-vivo studies have another carrier fluid, blood, which could influence the indicator dilution curve. As blood is a more viscous carrier fluid than water, the Reynolds number will decrease. As a result, the diffusion coefficient is expected to be lower. The diffusion coefficient influences the parameter λ of the LDRW model, which equals the Peclet number divided by 2. The Peclet number represents the ratio between diffusion and convection time. An increase in viscosity produces therefore an increase in λ, whereas μ, which is representative of the MTT, is not affected [25].

Our study may provide a good alternative for volume measurement in the perioperative setting and in critical care. The safety of SonoVue® has been investigated in different settings. In a large retrospective study on assessment of adverse events in 28 Italian Centers, serious adverse events were found in 0.0086% [26,27]. SonoVue® microbubbles are composed of SF6 gas with a phospholipid monolayer shell. The elimination of the SF6 gas via the lung is reported to be, even in patients with obstructive pulmonary disease and pulmonary fibrosis, in the same range as in healthy volunteers with a 80-90% clearance within 11 minutes [26]. The phospholipid monolayer is metabolized in the liver. These monolayers are commonly used in the formulation and manufacturing of liposomes, a drug delivery system that is approved by the United States Food and Drug Administration (FDA) and considered biologically safe [28]. Even in blunt abdominal trauma patients, the use of SonoVue® was proven to be safe [29]. Only in patients with a recent cardiac infarction or heart failure class III and IV, the European Medicines Agency (EMA) took precautions and these conditions are contraindicated for use of SonoVue®.

Limitations of our study relate to the thermodilution technique. Even with extended isolation of the set-up, volumes were overestimated using thermodilution. The pressure wires are normally used in coronary arteries to measure flow with thermodilution. These wires are very thin and ideal in our in-vitro set-up but not used for larger blood volume measurement. Thus, this correlation cannot be extrapolated in-vivo. Further research will be needed to compare CEUS and evaluate the margin of error with clinically used thermodilution methods for volume measurement, such as PiCCO® (Pulsion Medical Systems, Munich, Germany), and to estimate its value as a minimally-invasive and bedside-applicable technique in the ICU and operating room [3,30].

## Conclusions

CEUS seems a promising, minimally-invasive technique to measure volumes. Our in-vitro measurements showed a good correlation and level of agreement between CEUS volumes and the true volumes. We found a general overestimation of the measured volumes by the thermodilution technique and a general underestimation by CEUS, the latter being more evident for larger volumes and lower flows. This study suggests the use of CEUS to be superior to thermodilution and a good equivalent to the gold standard. We believe that this novel, minimally-invasive and non-nuclear method for measuring blood volume can be an asset in clinical research and practice. However, it is mandatory to validate this novel technique with frequently used techniques in daily, clinical practice.

## Competing interests

The authors declare that they have no competing interests.

## Authors’ contributions

IH developed the model together with EK, GR, TS and MM. IH and MM performed the image acquisitions. GR and MM performed the thermodilution measurements. MM and EK supervised the study and participated in the interpretation of the results. GR developed the fitting algorithm under supervision of MM. Analysis of the data was performed by IH. IH wrote the manuscript and made the plots. TS made Figure 1. MM revised the manuscript. PH revised the manuscript and advised with the statistical analysis. MV advised on the statistical analysis. HA revised the manuscript and analyzed the data. All authors read and approved the final manuscript.

## Supplementary Material

Additional file 1 **Transesophageal echography view of the inflow and outflow tube in the water-filled basin of the in-vitro set-up.** The change in acoustic intensity was analyzed with commercially available software (QLAB 8, Philips Healthcare, Andover, MA, USA). This software allows drawing of multiple regions of interest (ROIs) to obtain acoustic IDCs. Two ROIs were drawn within the thin polyurethane layer of the inflow and outflow tube. Click here for file

## References

[B1] Pinsky MR Payen D Functional hemodynamic monitoring Crit Care 2005 11 566 572 16356240 10.1186/cc3927 PMC1414021

[B2] Swan HJ Ganz W Forrester J Marcus H Diamond G Chonette D Catheterization of the heart in man with use of a flow-directed balloon-tipped catheter N Engl J Med 1970 11 447 451 5434111 10.1056/NEJM197008272830902

[B3] Sakka SG Ruhl CC Pfeiffer UJ Beale R McLuckie A Reinhart K Meier-Hellmann A Assessment of cardiac preload and extravascular lung water by single transpulmonary thermodilution Intensive Care Med 2000 11 180 187 10784306 10.1007/s001340050043

[B4] Reuter DA Huang C Edrich T Shernan SK Eltzschig HK Cardiac output monitoring using indicator-dilution techniques: basics, limits, and perspectives Anesth Analg 2010 11 799 811 20185659 10.1213/ANE.0b013e3181cc885a

[B5] Brands J Vink H Van Teeffelen JW Comparison of four mathematical models to analyze indicator-dilution curves in the coronary circulation Med Biol Eng Comput 2011 11 1471 1479 22095316 10.1007/s11517-011-0845-9 PMC3223587

[B6] Maddison B Wolff C Findlay G Radermacher P Hinds C Pearse RM Comparison of three methods of extravascular lung water volume measurement in patients after cardiac surgery Crit Care 2009 11 R107 19580649 10.1186/cc7948 PMC2750149

[B7] Mischi M Kalker TA Korsten EH Contrast echocardiography for pulmonary blood volume quantification IEEE Trans Ultrason Ferroelectr Freq Control 2004 11 1137 1147 15478975 10.1109/tuffc.2004.1334846

[B8] Mischi M Jansen AH Korsten HH Identification of cardiovascular dilution systems by contrast ultrasound Ultrasound Med Biol 2007 11 439 451 17280768 10.1016/j.ultrasmedbio.2006.09.013

[B9] Korsten HH Mischi M Grouls RJ Jansen A van Dantzig JM Peels K Quantification in echocardiography Semin Cardiothorac Vasc Anesth 2006 11 57 62 16703235 10.1177/108925320601000110

[B10] Schreiner BF Jr Murphy GW James DH Yu PN Pulmonary blood volume in patients with congestive heart failure Trans Assoc Am Physicians 1966 11 250 261 5333535

[B11] McGaff CJ Roveti GC Glassman E Milnor WR Pulmonary blood volume in rheumatic heart disease and its alteration by isoproterenol Circulation 1963 11 77 84 17894029 10.1161/01.cir.27.1.77

[B12] Wise ME Tracer dilution curves in cardiology and random walk and lognormal distributions Acta Physiol Pharmacol Neerl 1966 11 175 204 5336541

[B13] Bland JM Altman DG Statistical methods for assessing agreement between two methods of clinical measurement Lancet 1986 11 307 310 2868172

[B14] Shrout PE Fleiss JL Intraclass correlations: uses in assessing rater reliability Psychol Bull 1979 11 420 428 18839484 10.1037//0033-2909.86.2.420

[B15] Weir JP Quantifying test-retest reliability using the intraclass correlation coefficient and the SEM J Strength Cond Res 2005 11 231 240 15705040 10.1519/15184.1

[B16] Herold I Kuenen M Mischi M Korsten H Blood volume and ejection fraction measurements using CEUS Proceedings on the 15th European Symposium on Ultrasound Contrast Imaging 2010 55 56 Erasmus University Rotterdam

[B17] Mulvana H Stride E Hajnal JV Eckersley RJ Temperature dependent behavior of ultrasound contrast agents Ultrasound Med Biol 2010 11 925 934 20447756 10.1016/j.ultrasmedbio.2010.03.003

[B18] Goddi A Novario R Tanzi F Di Liberto R Nucci P In vitro analysis of ultrasound second generation contrast agent diluted in saline solution Radiol Med 2004 11 569 579 15195019

[B19] Vos HJ Single microbubble imaging Rotterdam 2010 Rotterdam: Erasmus University 131 145

[B20] Ugander M Kanski M Engblom H Gotberg M Olivecrona GK Erlinge D Heiberg E Arheden H Pulmonary blood volume variation decreases after myocardial infarction in pigs: a quantitative and noninvasive MR imaging measure of heart failure Radiology 2010 11 415 423 20656833 10.1148/radiol.10090292

[B21] Mischi M den Boer JA Korsten HH On the physical and stochastic representation of an indicator dilution curve as a gamma variate Physiol Meas 2008 11 281 294 18367805 10.1088/0967-3334/29/3/001

[B22] Mischi M van den Bosch HC den Boer JA Verwoerd J Grouls RJ Peels CH Korsten HH Intra-thoracic blood volume measurement by contrast magnetic resonance imaging Magn Reson Med 2009 11 344 353 19161145 10.1002/mrm.21824

[B23] Keller MW Segal SS Kaul S Duling B The behavior of sonicated albumin microbubbles within the microcirculation: a basis for their use during myocardial contrast echocardiography Circ Res 1989 11 458 467 2752551 10.1161/01.res.65.2.458

[B24] Tangelder GJ Slaaf DW Muijtjens AM Arts T oude Egbrink MG Reneman RS Velocity profiles of blood platelets and red blood cells flowing in arterioles of the rabbit mesentery Circ Res 1986 11 505 514 3802426 10.1161/01.res.59.5.505

[B25] Mischi M Kalker AA Korsten HH Cardiac image segmentation for contrast agent videodensitometry IEEE Trans Biomed Eng 2005 11 277 286 15709665 10.1109/TBME.2004.840500

[B26] Morel DR Schwieger I Hohn L Terrettaz J Llull JB Cornioley YA Schneider M Human pharmacokinetics and safety evaluation of SonoVue, a new contrast agent for ultrasound imaging Invest Radiol 2000 11 80 85 10639039 10.1097/00004424-200001000-00009

[B27] Piscaglia F Bolondi L The safety of Sonovue in abdominal applications: retrospective analysis of 23188 investigations Ultrasound Med Biol 2006 11 1369 1375 16965977 10.1016/j.ultrasmedbio.2006.05.031

[B28] Kang ST Yeh CK Ultrasound microbubble contrast agents for diagnostic and therapeutic applications: current status and future design Chang Gung Med J 2012 11 125 139 22537927 10.4103/2319-4170.106159

[B29] Cagini L Gravante S Malaspina CM Cesarano E Giganti M Rebonato A Fonio P Scialpi M Contrast enhanced ultrasound (CEUS) in blunt abdominal trauma Crit Ultrasound J 2013 11 1 S9 23902930 10.1186/2036-7902-5-S1-S9 PMC3711741

[B30] Linton RA Band DM Haire KM A new method of measuring cardiac output in man using lithium dilution Br J Anaesth 1993 11 262 266 8123404 10.1093/bja/71.2.262

